# Establishment of reference values of α-tocopherol in plasma, red blood cells and adipose tissue in healthy children to improve the management of chylomicron retention disease, a rare genetic hypocholesterolemia

**DOI:** 10.1186/s13023-016-0498-8

**Published:** 2016-08-12

**Authors:** Charlotte Cuerq, Lioara Restier, Jocelyne Drai, Emilie Blond, Adeline Roux, Sybil Charriere, Marie-Caroline Michalski, Mathilde Di Filippo, Emile Levy, Alain Lachaux, Noël Peretti

**Affiliations:** 1 Biochemistry Department, Lyon Sud Hospital, Hospices Civils de Lyon, Lyon, France; 2 INSERM U1060, INRA UMR 1397, INSA-Lyon, CarMeN Laboratory, Université Lyon 1, Lyon, France; 3 Pediatric Hepato-Gastroenterology and Nutrition Unit, Hôpital Femme Mère Enfant de Lyon, Hospices Civils de Lyon, Lyon, Bron France; 4 Hospices Civils de Lyon, Pole IMER, Lyon, France; 5 Fédération d’endocrinologie, maladies métaboliques, diabète et nutrition, Hôpital Louis Pradel, Hospices Civils de Lyon, Lyon, Bron France; 6 Dyslipidemia Unity, Department of Biochemistry and Molecular Biology, Centre de Biologie et de Pathologie Est, Hospices Civils de Lyon, Lyon, Bron France; 7 Research Centre, CHU Sainte-Justine, Université de Montréal, Montréal, Québec H3T 1C5 Canada; 8 Department of Nutrition, Université de Montréal, Montréal, Québec H3T 1A8 Canada

**Keywords:** α-tocopherol, Vitamin E, Red blood cells, Adipose tissue, Primary hypocholesterolemia, Anderson’s disease, Chylomicron retention disease, Reference interval

## Abstract

**Background:**

Chylomicron retention disease (CMRD), a rare genetic hypocholesterolemia, results in neuro-ophtalmologic damages, which can be prevented by high doses of vitamin E during infancy. In these patients, plasma vitamin E concentration is significantly reduced due to defects of chylomicron secretion. Vitamin E in adipose tissue (AT) and red blood cells (RBC) have been proposed as potential relevant biomarkers of vitamin E status but no reference values in children are available. The objectives were (i) to establish age-reference intervals in healthy children for α-tocopherol in plasma, red blood cells (RBC) and adipose tissue (AT) and (ii) to determine the variations of α-tocopherol in patients with CMRD after oral treatment with vitamin E.

**Methods:**

This prospective study included 166 healthy children (1 month - 18 years) and 4 patients with CMRD. Blood and AT were collected in healthy children during a scheduled surgery and in patients before and after a 4-month treatment with α-tocopherol acetate.

**Results:**

The reference ranges for α-tocopherol were 11.9 - 30 μmol/L in plasma, 2.0 - 7.8 μmol/L packed cells in RBC and 60 - 573 nmol/g in AT. α-tocopherol levels in plasma correlated with those of RBC (*r* = 0.31; *p* < 0.01).

In patients with CMRD after 4 months treatment, α-tocopherol concentrations remained less than 70 % of the control values in plasma, increased by 180 % to reach normal values in RBC, and remained stable in the normal range in AT.

**Conclusion:**

This study establishes pediatric reference intervals for α-tocopherol in plasma, RBC and AT. These values will be beneficial in assessing accurate α-tocopherol status in children and to optimize the monitoring of rare diseases such as CMRD. Our data suggest that RBC α-tocopherol, appears as a relevant biomarker to appreciate the effectiveness of treatment with α-tocopherol in patients with a rare primary hypocholesterolemia. The biopsy of AT could be used at diagnosis to assess the severity of the vitamin E deficiency and periodically after a long duration of vitamin E therapy to assess whether the treatment is effective, based on reference intervals defined in this study.

## Background

Vitamin E is known to be an essential micronutrient for promoting the development and maintaining the health due to its antioxidant and non-antioxidant biological activities [1, 2]. As a chain-reaction breaking antioxidant, vitamin E prevents the propagation of lipid-peroxidation, especially of polyunsattured fatty acids of cell membranes and low-density lipoproteins (LDL) from oxidation by free radicals. It also modulates genes expression and enzymatic activities and inhibits cell proliferation, platelet aggregation and monocyte adhesion [3]. Chylomicron Retention Disease (CMRD) (OMIM#246700), also called Anderson’s Disease (OMIM#607689), is a rare genetic disease due to mutations in the *SARA2* gene coding for the Sar1b GTPase protein [4]. It is among the genetic syndromes associated with congenital lipid malabsorption such as abetalipoproteinemia (ABL) and familial hypobetalipoproteinemia, a heterogeneous group of disorders characterised by a decrease of LDL-cholesterol (LDLc) and apolipoprotein B (Apo B) [5]. The clinical expression of these diseases is an important lipid malabsorption with severe malnutrition in the neonatal period along with ataxic neuropathy and ophthalmologic impairments during the second decade of life due to α-tocopherol deficiency [6]. In these patients, plasma vitamin E concentration is significantly reduced due to defects of chylomicron assembly and secretion, which are obligatory for its absorption. Similarly, the lack of low-density lipoproteins (LDL) and very-low-density lipoproteins (VLDL), necessary for its transport into the blood, are additional complicating factors. As plasma tocopherol is a poor indicator of body vitamin E stores in familial hypocholesterolemia, we needed an alternative measure less dependent on blood lipids for their monitoring. It has been estimated that about 90 % of the total body content of tocopherol is found in adipose tissue [7]. Therefore, vitamin E in adipose tissue (AT) has been proposed as a potential indicator of long-term intake of vitamin E. Studies performed in ABL patients have shown that treatment with large doses (10 g/d) of vitamin E prevented complications and increased tocopherol concentration in AT to normal levels [8, 9]. However, while some data on the concentration of vitamin E in AT are available in the literature pertinent to adults [10–12], there are no reference values for children. The age factor may influence the level of the micronutrients in healthy people as is the case for vitamin A [13, 14]. In addition, the AT biopsy remains an invasive procedure difficult to perform during the systematic management of children with primary hypocholesterolemia. An easier alternative could be to measure vitamin E levels in red blood cells (RBC) but the scientific literature is deficient in reference values for vitamin E levels in RBC in American and European children.

The objectives of our study were, therefore, first to establish reference intervals for α-tocopherol in plasma, RBC and AT in healthy children aged 1 month to 18 years, and second to determine whether the measurement of α-tocopherol in RBC could be an alternative to the AT biopsy to determine the vitamin E status in patients with CMRD.

## Methods

### Design and subjects

#### Healthy controls

Eligible subjects were healthy children aged 1 month to 18 years with a scheduled surgery. Children were recruited from the digestive or orthopedic surgical units of a university pediatric hospital. Exclusion criteria for the study were dyslipidemia, malnutrition, renal or hepatic diseases and vitamin E treatment. The study protocol was approved by the hospital ethics committee. The parents (and children when they were old enough) signed a non-opposition form. Patients were enrolled without regardless of race, gender or ethnic background. Children were divided into three age groups: 1 month to 2 years, 2 - 11 years, 12 - 18 years based on slightly modified international recommendations [15]. They were recruited from January 2009 to October 2011.

#### CMRD patients

Four patients with CMRD (3 girls and 1 boy, from 15 to 21 years, mean age = 19 years) were also included in the present study. All patients have been treated for several years by 50 mg/kg/d of α-tocopherol acetate. To evaluate the variation of α-tocopherol status, patients had a 2 month-washout period and they were then treated for 4 months with 50 mg/kg/d of tocopherol acetate. Alpha-tocopherol concentrations were measured in plasma, RBC and AT at the beginning and the end of the 4 months of treatment. The study protocol was approved by the hospital ethics committee and by the National Agency for Medicines and Health Products and complied with the Helsinki Declaration. If the child was a minor, a detailed informed consent form was signed by the child and his parents. When they were major, the children signed their own consent before their participation in the study.

### Sample collection and processing

#### Healthy controls

At the time of peripheral venipuncture, 6 ml of blood (3 mL sample in lithium heparin and 3 mL sample in additive clot activator tubes) were collected for determination of lipid profile and vitamin E in plasma and RBC. The tubes were processed within 4 hours after collection. At the beginning of surgery, three samples of approximately 100 mg of subcutaneous AT were collected for determination of vitamin E concentration in AT. Immediately, AT samples were washed with 1 mL saline solution, kept in a plastic adaptor, and frozen in liquid nitrogen and then stored < -70 °C.

#### CMRD patients

Before the beginning and at the end of the 4 months treatment with α-tocopherol acetate, 6 ml of blood were collected for determining the lipid profile and vitamin E concentrations in plasma and in RBC. After local anesthesia, two samples of approximately 50 mg of abdominal subcutaneous AT were collected by needle for determination of vitamin E in AT. Samples were processed as described for controls.

### High pressure liquid chromatography (HPLC) analysis of vitamin E

Plasma, erythrocytes and adipose tissue samples for determination of α-tocopherol concentrations were analyzed by HPLC using a Summit Dionex system (Thermo Fisher Scientific) and Chromeleon software (version 6.80, Thermo Fisher Scientific). Two levels of internal quality control were assayed at the beginning of each run. Furthermore, the laboratory participated regularly in external quality-assurance programs allowing monitoring of the measurement methods (bias and total error).

#### Plasma assay

Briefly, after precipitation of plasma proteins by ethanol, α-tocopherol was extracted into hexane, evaporated under nitrogen and the dried residue was dissolved in methanol/ethanol (85/15, v/v).

The eluate was analyzed by HPLC at 292 nm. Separation was carried out on an Adsorbosphere HS C18 3 mm (Interchim) held at 37 °C, using a gradient elution system starting with 100 % methanol-acetonitrile (40/60, v/v) and ending with a 100 % mixture of methanol-acetonitrile-dichloromethane (46/30/24, v/v) as described by Steghens et al [16]. Tocol was used as internal standard for measurement of vitamin E concentration to correct losses during liquid/liquid extraction. The intra-assay coefficients of variation (CV) for α-tocopherol in plasma were 2.4 and 1.1 % at respectively 17 and 40 μmol/L. The inter-assay CVs were respectively 3.9 % and 4 % at the same levels of concentration.

#### Erythrocytes assay

Erythrocytes were prepared for tocopherol analysis as previously described [17]. In brief, erythrocytes were washed 3 times in saline solution (9 g/L NaCl) containing pyrogallol (10 g/L) and resuspended in this solution to give a hematocrit of 50 % and 1 mL aliquots of washed erythrocytes were stored at < -70 °C until analysis. Alpha-tocopherol was analysed as described above. The intra-assay CV was 4.1 % at 5.3 μmol/L packed cells.

#### Adipose tissue assay

AT samples were removed from the connector, cut into smaller pieces, weighed (32.5 mg on average), transferred to a ground glass tissue grinder containing ethanol and an internal standard (Tissue Grind Comp Sz 20 Kontes Kimble-Chase LLC) and chilled on ice. The AT was ground and produced a uniform homogenate. The homogenate was transferred to a 7 ml glass tube and α-tocopherol was extracted by hexane as described above. The intra-assay CV was at 10.1 % at 460 nmol of α-tocopherol per gramme of adipose tissue.

### Measurement of serum lipids concentration

Total cholesterol, triglycerides and HDL-cholesterol were measured by routine laboratory procedures using an automated analyzer (Architect, Abbott Diagnostic). The inter-assay coefficient of variation was less than 3 % over the sample concentration range for these analytes. LDL-cholesterol was calculated using the Friedwald formula.

### Data analysis

The characteristics of healthy children and patients were reported using descriptive statistics (e.g. mean, standard deviation) for each age group and for all children. Reference intervals were established according to the Clinical and Laboratory Standards Institute (CLSI) and the International Federation of Clinical Chemistry (IFCC) guidelines on defining, establishing, and verifying reference (C28-A3) [18, 19]. Results were reviewed to detect outliers using the Tukey’s test prior to estimating reference intervals. Since the number of sample by age was less than the recommended 120, the non parametric percentile method was used to estimate reference intervals. Spearman’s rank correlations coefficients were calculated between AT, RBC plasma α-tocopherol concentration and cholesterol. Two-way analysis of variance (ANOVA) were performed to assess the influence of age and gender on α-tocopherol. Analyses were performed using SAS version 9.2 (SAS Institute) and MedCalc version 12.7.0.0 (MedCalc software).

## Results

### Healthy controls

**Fig. 1:**
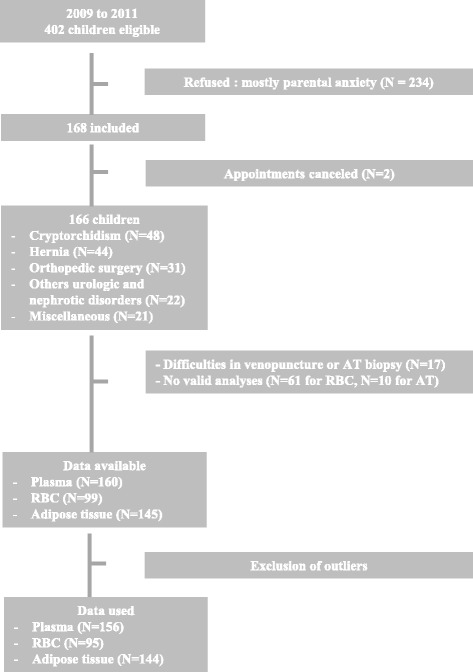
Flow chart explaining the sample size reduction. RBC: Red Blood Cells, AT: Adipose Tissue

**Fig. 2:**
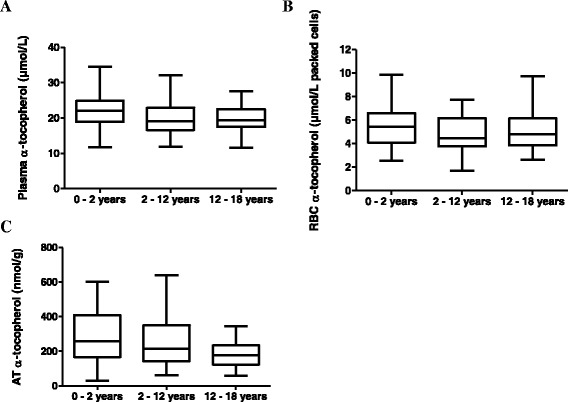
Box plot representation of α-tocopherol concentrations according to the age. Concentrations of vitamin E in **a** plasma, **b** red blood cells (RBC), **c** adipose tissue (AT). The line in the middle of the box is plotted at the median. The boxs represent from the 25th to 75th percentiles. The whiskers indicate the minimum and the maximum value

Of 402 eligible individuals, 234 (58 %) declined participation, mainly because of parental concern and 2 cancelled their appointment. Finally, 166 (73 females, 43 %) were included in the analysis. The flowchart for the healthy children is represented in Fig. 1. The main indications for surgery were a cryptorchidism (29 %) or a hernia repair (majority were inguinal or umbilical hernias) (27 %). The other interventions were orthopedic (19 %), urology and nephrology interventions (13 %) or miscellaneous (12 %). For the 166 subjects enrolled, the data were available: i) from 160 subjects for plasma tocopherol, ii) from 99 subjects for RBC tocopherol and iii) from 145 subjects for AT tocopherol for various reasons (difficulties in AT biopsy and venipuncture, or no valid analyses due to logistical and storage problems). Results were reviewed to detect outliers using Tukey’s test prior to analysing data, 4 outliers were excluded for plasma and RBC tocopherol analysis and 1 outlier was excluded for AT tocopherol analysis. The age and anthropometric characteristics of the study participants are described in Table 1. Mean and reference values for plasma, RBC and AT α-tocopherol concentrations by age-specific distribution are reported in Table 2. No difference attributable to sex was found in this study for α-tocopherol neither in plasma, RBC nor AT (data non shown). Mean plasma concentrations decreased slightly with age from 22.1 (children 1 month to 2 years) to 19.8 μmol/L (children 12 to 18 years) (*p* < 0.05) as well as mean AT α-tocopherol concentrations from 291 to 177 nmol/g (*p* < 0.01). RBC α-tocopherol concentrations were not influenced by age ranging from 4.67 to 5.47 μmol/L packed cells (p = NS) (Fig. 2).

**Table 1:** Physical and biochemical parameters of included healthy children and patients with Chylomicron Retention Disease (CMRD)

	Healthy children				CMRD
	0 - 2 years	2 - 12 years	12 - 18 years	Total	Total
n	56	75	35	166	4
Sex (F/M)	24/32	32/43	17/18	73/93	3/1
Age (y)	1.2 ± 0.6	5.9 ± 2.9	14.9 ± 1.7	6.2 ± 5.4	19 ± 2.8
Weight (kg)	9.5 ± 2.9	21.7 ± 9.1	55.3 ± 15.4	24.7 ± 19.3	54.0 ± 11.2
Height (cm)	74.7 ± 11.3	114.1 ± 19.0	163.0 ± 12.1	111.6 ± 35.4	160 ± 9.9
BMI (kg/m^2^)	16.85 ± 2.0	16.01 ± 1.9	20.4 ± 4.2	17.23 ± 3.1	20.2 ± 3.6
Total cholesterol (mmol/L)	3.74 ± 0.8	3.98 ± 0.8	3.95 ± 0.6	3.93 ± 0.7	2.31 ± 0.8
HDL cholesterol (mmol/L)	1.20 ± 0.5	1.31 ± 0.3	1.27 ± 0.2	1.28 ± 0.4	0.5 ± 0.1
LDL cholesterol (mmol/L)	2.24 ± 0.6	2.39 ± 0.6	2.28 ± 0.5	2.33 ± 0.6	1.50 ± 0.7
Triglycerides (mmol/L)	0.82 ± 0.5	0.63 ± 0.4	0.90 ± 0.5	0.74 ± 0.4	0.68 ± 0.3
Apolipoprotein B (g/L)	0.68 ± 0.2	0.66 ± 0.5	0.63 ± 0.1	0.65 ± 0.2	0.37 ± 0.2

Results as expressed as mean ± standard deviation

**Table 2:** Age-partitioned pediatric reference intervals for vitamin E in plasma, red blood cells (RBC) and adipose tissue

Test	Age (years)	Samples (n)	Mean	Lower limit	Upper limit
Plasma α-tocopherol (μmol/L)	0 - 2	48	22.1	11.8	34.2
	2 - 12	73	20	11.9	30.2
	12 - 18	35	19.8	11.6	27.6
	0 - 18	156	20.6	11.9	30
α-tocopherol/cholesterol ratio	0 - 18	113	5.27	3.3	7.1
α-tocopherol/triglycerides ratio	0 - 18	113	33.3	9.6	55.3
RBC α-tocopherol (μmol/L packed cells)	0 - 2	30	5.47	2.5	9.9
	2 - 12	37	4.67	1.7	7.8
	12 - 18	28	5.12	2.6	9.8
	0 - 18	95	4.96	2	7.8
Adipose tissue α-tocopherol (nmol/g)	0 - 2	48	291	37	597
	2 - 12	64	267	61	596
	12 - 18	32	177	58	344
	0 - 18	144	258	60	573

Plasma α-tocopherol correlated significantly with total cholesterol (*r* = 0.49, *p* < 0,001), LDLc (*r* = 0.42, *p* < 0,001) and to a lesser value with RBC α-tocopherol (*r* = 0.31, *p* < 0.01). Plasma and adipose α-tocopherol concentrations correlated only in the group of children aged 12 to 18 years (*r* = 0.35, *p* < 0.1). No correlation was found in this study between RBC and AT α-tocopherol (*r* = -0.02, p = NS).

### CMRD patients

As expected for individuals affected with CMRD, we observed lower concentrations of total cholesterol, LDLc, HDLc and Apo B in patients with CMRD compared with control subjects (Table 1).

After the wash out of 2 months and before the 4-month treatment period with α-tocopherol acetate, patients showed decreased plasma (med = 5.8 ± 3.7 μmol/L) and RBC (med = 2.1 ± 1.9 μmol/L packed cells) α-tocopherol concentrations. The median AT α-tocopherol concentration was 276 ± 227 nmol/g, close to the concentration found in healthy control children.

**Fig. 3:**
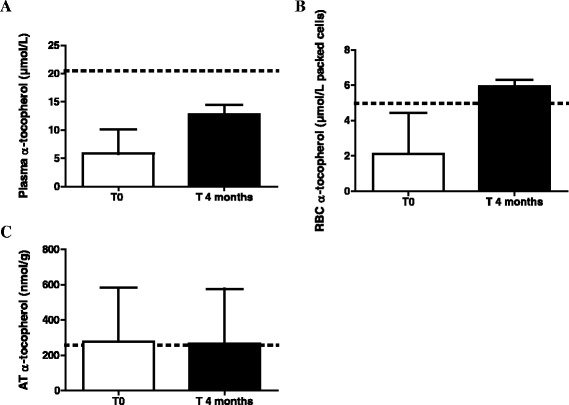
Evolution of vitamin E concentrations after 4 months treatment with α-tocopherol acetate in patients with CMRD. Concentrations of vitamin E in **a** plasma, **b** red blood cells (RBC), **c** adipose tissue (AT) after 4 months treatment with α-tocopherol acetate (50 mg/kg/d). CMRD : Chylomicron Retention Disease. Results are expressed as median with interquartile range. The horizontal line with the dotted lines represents the mean observed in healthy children

After the 4 month-treatment period, the median α-tocopherol concentration increased from 5.8 ± 3.7 to 14.1 ± 3.4 μmol/L in plasma but remained less than 70 % of the mean observed in the controls. However, RBC α-tocopherol increased by 180 % up to 5.9 ± 1.4 μmol/L packed cells in RBC close to the mean observed in controls. The median AT α-tocopherol concentration remained stable and normal at 266 ± 217 nmol/g (Fig. 3).

## Discussion

Only a few studies about antioxidant vitamins status for children have been carried out in the last decade in European countries and no data are available to our knowledge on the reference values of α-tocopherol in RBC and AT in American and European children. For the first time, this study i) establishes current reference intervals in children for the distribution of α-tocopherol in plasma, RBC and AT and provides information on the distribution of these variables by age in a cohort of French children from 1 month to 18 years ii) reports data on the concentration of vitamin E in CMRD patients in RBC and AT and their evolution during treatment.

### Plasma concentrations in healthy children/adolescents and in CMRD patients

In this prospective study of 166 healthy children, we reported mean plasma tocopherol concentrations and reference values very close to those observed previously in pediatric populations (Table 3) [13, 20, 21]. However, very few studies published reference intervals for very young children (<2 years old). Recently, Raizman et al. also reported that vitamin E levels rose within the first year of life and observed the widest interval within the first year of life assuming that the broad ranges could reflect changes over different gestational ages [14]. Values reported in adults were different than those in children, as shown by the SU.VI.MAX study, which reported mean α-tocopherol concentrations close to 31 μmol/L in a cohort of 12,741 French volunteers aged 35 to > 60 years [22].

**Table 3:** Table summarizing our data and those obtained in previous studies

	Our study	Studies in children	Studies in adults
Plasma α-tocopherol	0 - 2 yr (n = 48): 22.1 μmol/L	0 - <1 yr canadian children (*n* = 85): 5 - 50 μmol/L [14]	35 - 45 yr french females (*n* = 2163): 29.8 ± 6.7 μmol/L [22]
			35 - 45 yr french males (*n* = 138): 31.9 ± 5.5 μmol/L [22]
		1 - <19 yr canadian children (*n* = 245): 14.5 - 33 μmol/L [14]	45 - 50 yr french females (*n* = 1499): 31.0 ± 6.6 μmol/L [22]
			45 - 50 yr old french males (*n* = 1379): 31.3 ± 6.7 μmol/L [22]
	2 - 12 yr (n = 73): 20 μmol/L	10 - 15 yr french males and females (*n* = 263 and 246): ≈ 20 and 21 μmol/L [20]	50 - 60 yr old french females (*n* = 1645): 31.5 ± 7.2 μmol/L [22]
			50 - 60 yr old french males (*n* = 1919): 32.5 ± 7.4 μmol/L [22]
	12 - 18 yr (n = 35): 19.8 μmol/L	12.5 - 17.49 yr european adolescents (*n* = 1053): 23 μmol/L [21]	> 60 yr french females (*n* = 127): 33.2 ± 7.5 μmol/L [22]
			> 60 yr french males (*n* = 159): 32.7 ± 9.3 μmol/L [22]
RBC α-tocopherol	0 - 2 yr: 5.47 μmol/L packed cells	3 - 6 yr : 4.65 μmol/L packed cells [33]	4.09 μmol/L packed cells [33]
	2 - 12 yr: 4.67 μmol/L packed cells	7 - 10 yr : 4.07 μmol/L packed cells [33]	5.1 μmol/L packed cells [35]
	12 - 18 yr: 5.12 μmol/L packed cells	11 yr: 4.09 μmol/L packed cells [33]	18.5 nmol/g Hb (≈ 6.60 μmol/L packed cells if Hb = 150 g/L and haematocrit 0.42) [34]
Adipose tissue α-tocopherol	0 - 2 yr: 291 nmol/g AT	None	192 μg/g fatty acids (≈ 292 nmol/g AT) [12]
	2 - 12 yr: 267 nmol/g AT		190 nmol/g AT in 347 males and 286 nmol/g in 111 females [11]
	12 - 18 yr: 177 nmol/g AT		199 nmol/g AT [10]

Vitamin E is transported in circulation by plasma lipoproteins mainly LDL and HDL [23, 24]. Mean cholesterol values reported in this study were closed to those reported in European children and adolescents in the HELENA and IDEFICS studies [25, 26] and slightly lower than those reported among French children [27–29]. Since α-tocopherol is known to increase with the degree of hyperlipidemia and is dependent on lipid metabolism for delivery to tissues, adjustment for total cholesterol or lipids is also important to assess vitamin E status in some special conditions such as cholestasis or cystic fibrosis [24, 30]. The observed vitamin E/lipid ratios observed in this study are in agreement with previous findings [14, 24, 31].

In CMRD patients, we confirmed that, even they were treated with α-tocopherol acetate 50 mg/kg/d, plasma α-tocopherol concentrations remained decreased because its absorption is impaired and lipoprotein transport pathways are saturated. Even if the α-tocopherol/cholesterol ratio reached normal values at the end of treatment, this ratio does not seem to be ideal since α-tocopherol and cholesterol remain both lower than normal values. It is therefore an inaccurate “normal value” since the capacity to transport adequate quantity of vitamin E to tissues remains impaired [32].

### RBC concentrations in healthy children/adolescents and in CMRD patients

Our results regarding RBC α-tocopherol are consistent with those previously described, although concentrations cannot simply be compared to other studies because these concentrations can be expressed in different ways. To our knowledge, a single Japanese study reported references values in children. Their data are in agreement with ours as they reported that the mean RBC tocopherol was 179 μg/100 mL packed cells, ranging from 79 to 320 μg/100 ml (corresponding to 4.16 μmol/L packed cells, range 1.84 – 7.41 μmol/L) in their 261 apparently healthy children aged 3 to 16 years old (For concentrations detailed by age, see Table 3) [33].

The concentration of vitamin E in RBC in adults seems to be equivalent to concentrations in children, given that RBC tocopherol was : i) 4.09 μmol/L packed cells in 26 adult controls aged between 20 and 33 years old in the study about Japanese children [33] ii) 18.5 nmol/g Hb (corresponding to 6.59 μmol/L packed cells if Hb = 150 g/L and haematocrit 0.42) in a control population of 67 healthy adults [34] iii) 5.1 μmol/L packed cells in a population of 20 healthy adults not supplemented [35].

In CMRD patients, the vitamin E in RBC was decreased to about half of the normal value defined in the study after 2 months of wash out but increased to a normal value after 4 months of supplementation. These results are consistent with the half-life of erythrocytes of ≈ 120 days and with results found by others in patients with genetic hypocholesterolemia : i) Bieri et al. reported normal concentrations of α-tocopherol in RBC in two abetalipoproteinemia patients that were administered 750 mg aqueous d-α-tocopherolyl succinate daily for several months [36] and ii) Hatam et al. reported on a single supplemented abetalipoproteinemia patient with RBC α-tocopherol concentrations ten times higher than a subject not taking supplemental vitamin E (although the RBC concentration was less than seen in normal subjects) [37]. Similarly, Clarke et al. observed that normal RBC α-tocopherol concentrations were noted in 2 homozygous hypobetalipoproteinemia patients receiving supplementation, whereas they remained low in one patient with ABL even if he was supplemented [38].

### AT concentrations in healthy children/adolescents and in CMRD patients

Since vitamin E is a fat-soluble compound stored in AT, concentrations in AT may be a better indicator of long term intake than plasma levels. One objective of this work was to establish reference values for α-tocopherol in AT in healthy children. We found that adipose α-tocopherol concentrations in children (258 ± 146 nmol/g, *n* = 144) were similar to those described in adults.

In adults, the concentration of α-tocopherol in AT was i) 192 μg/g fatty acids (corresponding to approximately 292 nmol/g of adipose tissue) in 727 healthy controls enrolled in the EURAMIC study [12] and ii) 190 nmol/g in 347 male and 286 nmol/g in 111 female controls [11]. Traber et al. who studied the effect of thermal injury on body stores of vitamin E in pediatric patients (*n* = 8, mean age = 15 years) showed that the AT α-tocopherol concentrations in the initial biopsy within the first week after injury were 199 nmol/g and reported that the normal range observed in healthy adults was 200 to 700 nmol/g [10]. Interestingly, as with plasma tocopherol, adipose α-tocopherol concentrations were significantly higher in very young children (<2 years old) than in older children (>12 years).

Finally, our CMRD patients who have been treated by the recommended daily dose of 50 mg/kg of tocopherol acetate for many years, had normal adipose α-tocopherol concentrations after 2 months of wash out and 4 months of treatment. This is in agreement with findings that some ABL patients on massive supplementation with vitamin E did achieve normal concentrations of AT tocopherol [8, 39] and illustrates that changes in AT are very slow. Handelman et al. estimated that more than 2 years are required for the α-tocopherol/γ-tocopherol ratio to reach a new steady state after a change in α-tocopherol intake [40].

### Correlations between α-tocopherol in plasma, RBC and AT in healthy children

Informations on the relationship between plasma, RBC and AT levels of α-tocopherol in the free-living human population are very limited.

We found a weak correlation between plasma and RBC α-tocopherol in children. This correlation coefficient was a bit lower than those reported by Mino et al. (*r* = 0.59, *p* < 0.001) [33] and Lehmann et al. (*r* < 0.40) [35] and was not improved by adjusting for lipids.

In this work, the coefficient of correlation between plasma and adipose α-tocopherol concentrations in the group of children aged 12 to 18 years old (*r* = 0.32, *p* = 0.06) was very close to those observed in adults by Kardinaal et al. (*r* = 0.31, *p* < 0.01) from 85 healthy, non-smoking volunteers aged 50–70 years in the Netherlands [41] and El-Sohemy et al. (*r* = 0.27, p not specified) in a population of 482 Costa Rican [11]. Su et al. also reported a weak correlation (*r* = 0.39, *p* < 0.01) between plasma and AT α-tocopherol concentrations in the control group of the EURAMIC study [42]. Finally, in a study with a small number of subjects (*n* = 20), Schäefer et al. showed that total tocopherol in plasma correlated significantly with AT total tocopherol (*r* = 0.47, *p* < 0.05) and that the content of n-3 fatty acids in AT influenced AT vitamin E negatively [43]. Taken together this indicates that adipose α-tocopherol concentrations are also influenced by factors other than α-tocopherol intake or blood concentrations such as genetic differences, needs for rapid development and growth, or overall dietary habits in children.

We failed to detect any correlation between AT and RBC in healthy controls subjects. Those results are consistent with the fact that AT α-tocopherol probably represents a long-term measure of α-tocopherol status whereas RBC α-tocopherol reflect a shorter-term intake.

## Conclusion

We established for the first time the reference values of α-tocopherol in plasma, RBC and AT in children, from the neonatal period to early adulthood. These values will be useful to assess the vitamin E nutritional status of children in epidemiological studies or in particular diseases, such as primary hypocholesterolemia including CMRD, familial hypobetalipoproteinemia and abetalipoproteinemia. Our data suggest that RBC α-tocopherol is a relevant biomarker to monitor children with chylomicron retention disease. It appears to be an appropriate biomarker of deficiency and effectiveness of treatment since it decreases rapidly and can be normalized after 4 months of supplementation; It is less influenced by lipids than plasma α-tocopherol. The biopsy of AT could be used at diagnosis to assess the severity of the vitamin E deficiency and periodically after a long duration of vitamin E therapy to assess whether the treatment is effective and achieves values close to normal. However, AT biopsies are not useful for regular monitoring due to the very slow change of vitamin E concentrations in AT.

Considering that platelet tocopherol concentrations are not influenced by lipids [35], reference values in children would have been relevant given that it is important to use more than one biomarker for tocopherol to evaluate the effectiveness of treatment in patients with severe hypocholesterolemia. Moreover, it is not clearly established whether increased oxidative stress is seen in genetic hypocholesterolemia. The measurement of urinary F2-isoprostanes in healthy children and CMRD patients would probably have provided valuable information [44].

## Abbreviations

Apo B, Apolipoprotein B; AT, Adipose tissue; CMRD, Chylomicron Retention Disease; HDL, High-density lipoproteins; LDL, Low-density lipoproteins; LDLc, LDL-cholesterol; NS, Non significant; RBC, Red blood cells.
